# Nanomaterials Toxicity and Cell Death Modalities

**DOI:** 10.1155/2012/167896

**Published:** 2012-12-05

**Authors:** Daniela De Stefano, Rosa Carnuccio, Maria Chiara Maiuri

**Affiliations:** ^1^Dipartimento di Farmacologia Sperimentale, Facoltà di Scienze Biotecnologiche, Università degli Studi di Napoli Federico II, Via D. Montesano 49, 80139 Napoli, Italy; ^2^INSERM U848, IGR, 39 Rue C. Desmoulins, 94805 Villejuif, France

## Abstract

In the last decade, the nanotechnology advancement has developed a plethora of novel and intriguing nanomaterial application in many sectors, including research and medicine. However, many risks have been highlighted in their use, particularly related to their unexpected toxicity *in vitro* and *in vivo* experimental models. This paper proposes an overview concerning the cell death modalities induced by the major nanomaterials.

## 1. Introduction

**Figure 1:**
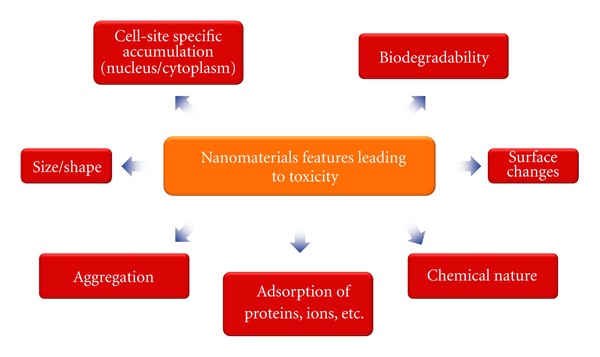


Nanotechnologies are emerging for important new applications of nanomaterials in various fields. Nanomaterials are defined as substances which have one or more external dimension in the nanoscale (1–100 nm). Nanomaterials, especially nanoparticles and nanofibres, show higher physical and chemical activities per unit weight. These properties explain their large application not only in industry but also in the scientific and medical researches. In fact, in these areas, the use of many kinds of manufactured nanoparticles products is in development, such as metal oxide nanoparticles (cerium dioxide, cupric oxide, titanium dioxide, zinc oxide, etc.), metal nanoparticles (gold, silver, platinum, palladium, etc.), C60 fullerenes nanocrystals, carbon nanotubes (CNTs), and quantum dots. Initially, the nanomaterials were believed to be biologically inert, but a growing literature has highlighted the toxicity and potential risks of their use. Extrapolations from the field of toxicology of particulate matter (less than 10 nm) confirm that nanoparticles present a range of harmful effects [1, 2]. In most cases, enhanced generation of reactive oxygen species (ROS), leading to oxidative stress which in turn may trigger proinflammatory responses, is assumed to be responsible for nanomaterials toxicity, although nonoxidative stress-related mechanisms have also been recently reported (see the extensive and interesting reviews [3–10]). However, despite intensive investigations, the understanding of nanomaterials-induced cellular damage remains to be clarified. The literature in the field suggests correlations between different physicochemical properties and the biological and toxicological effects of cells and tissues exposure to nanomaterials. First of all, nanomaterials are characterized by high specific surface area that correlates with high interfacial chemical and physical reactivity that, in turn, translates to biological reactivity [11]. The addition of different types of nanoparticles to various primary cell cultures or transformed cell lines may result in cell death or other toxicological outcomes, depending on the size of the nanomaterial. Quantum dots were reported to localize to different cellular compartment in a size-dependent manner [12]. Silica nanoparticles (40–80 nm) can enter into the nucleus and localize to distinct subnuclear domains in the nucleoplasm, whereas thin and coarse ones located exclusively in the cytoplasm [13]. Gold nanocluster (1.4 nm) intercalates within the major groove of DNA and is a potent inducer of cell death in human cancer cells [14]. Growing evidence suggests that the state of nanoparticles aggregation cannot be ignored; in fact, the toxicity may depend on the size of the agglomerate and not on the original nanoparticle size itself [15, 16]. For example, in rats exposed by inhalation to 20 nm or 250 nm titanium dioxide (TiO_2_) particles, the half-times for alveolar clearance of polystyrene test particles were proportional to the TiO_2_ particle surface area per million of macrophages [17, 18]. Clearly, a surface impurity, resulting from air or water contaminants such as bacterial endotoxin, could contribute to the cellular responses induced by nanomaterials, in particular immunological responses [16]. The same consideration is true for residual materials (surfactants or transition metals) arising from the synthetic process [6, 19, 20]. Nevertheless, the adsorption ability and surface activity are also involved in cellular influences of nanomaterials. When dispersed in culture medium, some metal oxide nanoparticles and CNTs could adsorb proteins, often called “protein corona” such as serum albumin, or calcium, which could change the biological activity of nanomaterials. This adsorption could be particle size and time dependent. In these conditions, many nanoparticles form secondary particles, which are a complex of nanoparticles and medium components [21–26]. For example, adsorbed albumin on the CNT was involved in phagocytosis of the macrophage via scavenger receptor [27]. A surface-engineered functionalization also may be linked with the biological nanomaterials activity, although in this item that is a wanted effect. Moreover, examples of dose-dependent toxicity also are evaluated [6, 28, 29]. As pointed out in a recent review [6], the degree of recognition and internalization of nanomaterials likely influences their distribution and may determine also their toxic potential. It has been reported that the number of internalized quantum dots (the intracellular dose) correlates with the toxicity in human breast cancer cell line [30]. Furthermore, the toxicity and cell death fate appear to correlate with the type of crystal structures [16, 31]. Finally, the nanomaterials degradability should also be taken into account (Figure 1). Nondegradable nanomaterials can accumulate into the cells and/or organs and exert damage effect as well as their degradation products [32–34]. However, it is not yet clear which of these parameters mainly influences the nanomaterials toxicity or if all of these features act together [35]. It is important to note that in the literature conflicting results are present. These are likely caused by variations in type, composition, size, shape, surface charge, and modifications of nanoparticles employed; use of various *in vivo* and *in vitro* models (the cell death mode may be also cell type dependent); experimental procedures (different methods to evaluate cell death; nanomaterials dose, concentrations and efficiency of cellular uptake, and time of exposure). This paper aims to give a critical overview concerning the different cell death modalities induced by nanomaterials.

Deregulated cell death is a common element of several human diseases, including cancer, stroke, and neurodegeneration, and the modulation of this cellular response can be an optimal target for an effective therapeutic strategy. Many cytotoxic agents are potent anticancer therapeutics, whereas cytoprotective compounds may be used to elude unwanted cell death in the context of stroke, myocardial infarction or neurodegenerative disorders [36, 37]. The complex molecular mechanisms and signalling pathways that control cell death are increasingly becoming understood, and it is now clear that different cell death subroutines play a critical role in multiple diseases. In many instances, the modality by which cells die is crucial to the cell death achievement at the organism level. The Nomenclature Committee on Cell Death (NCCD) has recently formulated a novel systematic classification of cell death based on morphological characteristics, measurable biochemical features and functional considerations [38]. We will consider these definitions of cell death in order to summarize and organize the molecular mechanisms underlying the nanomaterials toxicity. We could not report all the studies, and we apologize for this; we will describe the most recently, accurate, and representative ones in term of the described molecular mechanisms.

## 2. Nanomaterials and Apoptosis

Apoptosis is a form of cellular suicide that can be classified into *extrinsic* and *intrinsic* apoptosis. Extrinsic apoptosis indicates the cell death, caspase dependent, stimulated by extracellular stress signals that are sensed and propagated by specific transmembrane receptors. Three major lethal signalling cascades have been reported: (i) death receptor signalling and activation of the caspase-8 (or -10) and then caspase-3 cascade; (ii) death receptor signalling and activation of the caspase-8 then BH3-interacting domain death agonist (BID), mitochondrial outer membrane permeabilization (MOMP), caspase-9 and caspase-3 pathways; and (iii) ligand deprivation-induced dependence receptor signalling followed by (direct or MOMP-dependent) activation of the caspase-9 and after caspase-3 cascade [38]. Intrinsic apoptosis can be triggered by a plethora of intracellular stress conditions, such as DNA damage, oxidative stress, and many others. It results from a bioenergetic and metabolic catastrophe coupled to multiple active executioner mechanisms. This process could be caspase-dependent or- independent and is mediated by MOMP associated with the generalized and irreversible dissipation of the mitochondrial transmembrane potential, release of mitochondrial intermembrane space proteins into the cytosol (and their possible relocalization to other subcellular compartments), and the respiratory chain inhibition [38]. Apoptosis plays a fundamental role in development and for maintenance of tissue homeostasis in the adult organism. In addition, impairment of apoptosis may contribute to tumour progression.

Nanomaterials are described as triggers of extrinsic and intrinsic apoptotic pathways; however, the oxidative stress paradigm of nanomaterials-induced cell death linked to intrinsic apoptotic network is by far the most accepted, in fact many *in vitro* studies have identified increased ROS generation as an initiating factor of toxicity in nanomaterials exposed cells [3, 6, 7, 10, 39]. Although it is well established that the mode of cell death depends on the severity of the cellular insult (which may, in turn, be linked to mitochondrial function and intracellular energy), it has been difficult to set up a comprehensive mechanism of nanomaterials cell death based on conflicting observations present in the literature. Furthermore, in most of the studies, the molecular mechanisms underlying cell death are not investigated. Finally, another problem is the nonhomogeneity of the studies, in terms of materials and experimental methods used, which makes it difficult to compare.

Sarkar and colleagues showed that the nano-copper induces intrinsic apoptotic cell death in mice kidney tissue (*via* the increase of ROS and reactive nitrogen species production, regulation of Bcl-2 family protein expression, release of cytochrome *c* from mitochondria to cytosol, and activation of caspase-3), but, in addition, they observed the activation of FAS, caspase-8, and tBID, suggesting also the involvement of extrinsic pathways [40]. The exposure to nano-copper dose-dependently caused oxidative stress and led to hepatic dysfunction *in vivo*. Nano-copper caused the reciprocal regulation of Bcl-2 family proteins, disruption of mitochondrial membrane potential, release of cytochrome *c*, formation of apoptosome, and activation of caspase-3. These results indicate that nano-copper induces hepatic dysfunction and cell death via the oxidative stress-dependent signalling cascades and mitochondrial event [41].

Metallic nickel nanoparticles induced apoptotic cell death through an FAS/caspase-8/BID mediated, cytochrome *c*-independent pathway in mouse epidermal cells [42]. Nickel oxide nanoparticles excited in dose-dependent manner the increase of ROS production, lipid peroxidation, and caspase-3 activation in human airway epithelial and breast cancer cells [43]. Moreover, nickel ferrite nanoparticles provoked apoptosis in human lung epithelial cells through ROS generation via upregulation of p53 and Bax as well as the activation of caspases cascade [44].

*In vitro*, silicon dioxide (SiO_2_) nanoparticles increased ROS and RNS (reactive nitrogen species) production that, in turn, can induce the intrinsic apoptotic machinery [45]. Furthermore, Wang and collaborators showed that p53 plays a key role in silica-induced apoptosis *in vitro* (mouse preneoplastic epidermal cells and fibroblasts) and *in vivo* (p53 wild-type and deficient mice) [46].

TiO_2_ nanoparticles, sized less than 100 nm, triggered apoptotic cell death through ROS-dependent upregulation of FAS and activation of Bax in normal human lung fibroblast and breast epithelial cell lines [47]. Moreover, it was also demonstrated that TiO_2_ nanoparticles induced apoptosis through the caspase-8/BID pathway in human bronchial epithelial cells and lymphocytes as well as in mouse preneoplastic epidermal cells [48, 49]. Some reports indicated that TiO_2_ induced also lipid peroxidation, p53-mediated damage response, and caspase activation [50, 51]. In contrast, there are also reports demonstrating that TiO_2_ nanoparticles did not induce oxidative stress on mouse macrophages [52] as well as did not shown cytotoxicity in human dermal fibroblasts and lung epithelial cells [31].

A number of studies have been published concerning the effects of CNTs on apoptosis. Multiwall carbon nanotubes (MWCNTs) induced an increase of ROS, cell cycle arrest, decrease in mitochondrial membrane potential, determining apoptosis in different *in vitro* models [53–56]. In contrast, another study reported that these nanotubes were nontoxic [57]. Accordingly, it has been observed that MWCNTs did not stimulate cell death *in vitro* after acute exposure and neither after the continuous presence of their low amounts for 6 months [58]. Instead, apoptotic macrophages have been observed in the airways of mice after inhalation of SWCNTs (single-walled carbon nanotubes) [6]. Accordingly, several studies *in vivo* suggest that the exposure to SWCNTs leads to the activation of specific apoptosis signalling pathways [59, 60]. For more details, recent interesting reviews focus on the nanomaterials toxicity *in vivo* studies [6, 34].

Nanoparticles are frequently detected in lysosomes upon internalization, and a variety of nanomaterials have been associated with lysosomal dysfunction [61]. It has been established that lysosomal destabilization triggers the mitochondrial pathway of apoptosis [62, 63]. Carbon nanotubes were shown to induce lysosomal membrane permeabilization and apoptotic cell death in murine macrophages and human fibroblasts [64, 65]. Carbon black nanoparticles elicited intrinsic apoptosis in human bronchial epithelial cells with activation of Bax and release of cytochrome *c* from mitochondria, whereas TiO_2_ nanoparticles induced apoptosis through lysosomal membrane destabilization and cathepsin B release, suggesting that the pathway of apoptosis differs depending on the nanomaterials chemical nature [66]. The lysosomal destabilization induced by TiO_2_ is also confirmed in mouse fibroblasts [67]. SiO_2_ and several cationic nanoparticles, such as cationic polystyrene nanospheres and cationic polyamidoamine (PAMAM) dendrimers, have also shown the same mode of action [68–70]. However, also the micromaterials are able to destabilize lysosomes, in fact silica microparticles have been demonstrated to induce apoptosis in mouse alveolar macrophages by this molecular mechanism [70]. A comparative study of nano- versus microscale gold particles demonstrated that nanoparticles present a higher potency in the induction of lysosomal membrane destabilization [71].

Chronic or unresolved endoplasmic reticulum (ER) stress can also cause apoptosis [72, 73]. Zhang and colleagues reported that the ER stress signalling is involved in silver nanoparticles-induced apoptosis in human Chang liver cells and Chinese hamster lung fibroblasts [74]. Using *omic* techniques and systems biology analysis, Tsai and collaborators demonstrated that upon ER stress, cellular responses, including ROS increase, mitochondrial cytochrome *c* release, and mitochondria damage, chronologically occurred in the gold nanoparticles-treated human leukemia cells. This treatment did not induce apoptosis in the normal human peripheral blood mononuclear cells [75]. It has been shown that poly(ethylene glycol)-phosphoethanolamine (PEG-PE), an FDA-approved nonionic diblock copolymer widely used in drug delivery systems, accumulated in the ER and induced ER stress and apoptosis only in cancer cells (human adenocarcinomia alveolar basal epithelial), whereas it did not have effect in normal cells (secondary human lung fibroblasts and embryonic kidney cells) [76].

The predisposition of some nanoparticles to target mitochondria, ER, or lysosomes and initiate cell death could be used as a new cancer chemotherapy principle.

Interestingly, nanoparticles (polystyrene nanoparticles of 20–40 nm with two different surface chemistries, carboxylic acid, and amines) may also induce apoptosis in individual cells (differentiated human colorectal adenocarcinoma) that then propagates to other neighbouring cells through a “bystander killing effect.” The authors of this study suggest that ingested nanoparticles represent a potential health risk due to their detrimental impact on the intestinal membrane by destroying their barrier protection capability over time [77].

**Figure 2:**
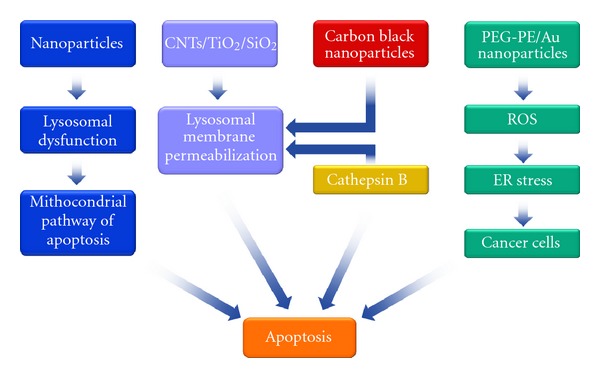


Surely in this context, a common incentive to synchronize the studies and research efforts is needed. The understand why cancer cells and distinctive normal cells have different cell fates as a result of nanomaterials exposure, focusing on the underlying mechanisms, will allow a better prediction of the consequences of exposure to nanomaterials and a safer assessment of the risks (Figure 2).

## 3. Nanomaterials and Mitotic Catastrophe

Recently, Vitale and colleagues suggested a novel definition of mitotic catastrophe based on functional consideration [78]. They proposed to consider mitotic catastrophe not a “pure” cell death executioner pathway but as an oncosuppressive mechanism that is triggered by perturbations of the mitotic apparatus, is initiated during the M phase of the cell cycle, is paralleled by some degree of mitotic arrest, and induces cell death (apoptosis or necrosis) and senescence [78].

**Figure 3:**
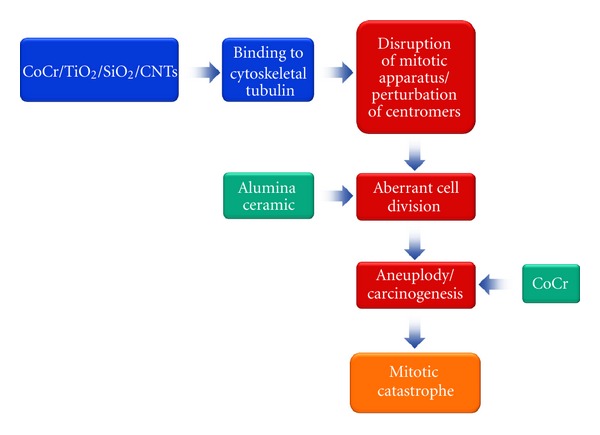


It has been reported that several nanomaterials, such as SiO_2_, TiO_2_, cobalt-chrome (CoCr) metal particles, and carbon nanotubes, interact with structural elements of the cell, with an apparent binding to the cytoskeleton and in particular the tubulins [79, 80]. In this setting, some evidence *in vitro* demonstrated that carbon nanotubes mimic or interfere with the cellular microtubule system, thereby disrupting the mitotic spindle apparatus and leading to aberrant cell division [81–83]. In particular, the perturbation of centrosomes and mitotic spindles dynamics caused by these nanoparticles results in monopolar, tripolar, and quadripolar divisions, that, in turn, could determinate aneuploidy [78], an event closely linked to the carcinogenesis. Tsaousi and collaborators found that alumina ceramic particles increase significantly in micronucleated binucleate cells [84], which is considered a morphological marker of mitotic catastrophe [78]. Interestingly, this increase was much greater after exposure of primary human fibroblasts to CoCr metal particles, suggesting that these nanoparticles are particularly efficient in affecting the mitotic machinery [84]. Apparently, the genotoxic effect of CoCr nanoparticles is size dependent. Indeed, CoCr nanoparticles induced more DNA damage than microsized ones in human fibroblasts (Figure 3). In fact, the mechanism of cell damage appears to be different after nano- or microparticles exposure. The enhanced oxidative DNA damage by the microparticles may result from a stronger ability of large particles to activate endogenous pathways of reactive oxygen species formation, for example, involving NADPH oxidases or mitochondrial activation. It also suggests that the observed genotoxic effect of the nanoparticles in the comet assay and the micronucleus assay (i.e., stronger aneugenic effect) is due to mechanisms other than oxidative DNA attack. A different mechanism of DNA damage by nanoparticles and microparticles is further suggested by measures of DNA damage from the comet and micronucleus assays. The comet assay revealed more damage in nanoparticle-exposed than in microparticle cells. In contrast, the micronucleus assay revealed slightly less centromere-negative micronuclei in nanoparticle exposed than in microparticle-exposed cells. This assay measures clastogenic, that is, double strand breakage events. Although some micronuclei in nanoparticle-exposed cells might not have been seen as a result of inhibition of cell division from greater cytotoxicity, these results point to a greater complexity of DNA damage caused by exposure to nanoparticles compared to microparticles [85]. A genotoxic effect has also described for silver nanoparticles that induced chromosomal aberrations, damage of metaphases, and aneuploidy in medaka (Oryzias latipes) cell line [86].

Further studies are needed to validate this dangerous potential effect of the nanomaterials. Obviously, close attention to safety issues will be required, also in the light of the potential interference between engineered nanomaterials and the environment.

## 4. Nanomaterials and Autophagy or “Autophagic Cell Death”

Autophagy is a highly conserved homeostatic process, involved in the recognition and turnover of damaged/aged proteins and organelles. During autophagy, parts of the cytoplasm are sequestered within characteristic double- or multi-membraned autophagic vacuoles (named autophagosomes) and are finally delivered to lysosomes for bulk degradation. This process is dynamically regulated by ATG (Autophagy-related gene) gene family and is finely controlled by several signalling pathways [87]. Autophagy constitutes a cytoprotective response activated by cells in the challenge to cope with stress. In this setting, pharmacological or genetic inhibition of autophagy accelerates cell death. On the basis of morphological features, the term “autophagic cell death” has widely been used to indicate instances of cell death that are accompanied by a massive cytoplasmic vacuolization [38]. The expression “autophagic cell death” is highly prone to misinterpretation and hence must be used with caution, but, discussion this problem is beyond the scope of this paper, and an excellent paper concerning this subject has been published [88]. In any case, “autophagic cell death” is used to imply that autophagy would execute the cell demise. In the literature, it has been reported that several classes of nanomaterials induce elevated levels of autophagic vacuoles in different animals and human cell culture as well as *in vivo* models (masterfully summarized in two recent reviews [10, 61]). Such nanomaterials include alumina, europium oxide, gadolinium oxide, gold, iron oxide, manganese, neodymium oxide, palladium, samarium oxide, silica, terbium oxide, titanium dioxide, ytterbium oxide, and yttrium oxide nanoparticles; nanoscale carbon black; fullerene and fullerene derivate; and protein-coated quantum dots. The induction of autophagy was evaluated using panoply of established methods, including the electron microscopy detection of autophagic vacuoles, the immunoblot detection of ATG expression level and/or LC3-I to LC3-II conversion (an established marker of autophagy activity) and/or cellular immunolabeling of punctate LC3-II in cytoplasmic vacuoles. These studies were performed *in vivo* but mainly in primary cells and/or cell lines from rat (alveolar macrophages, kidney, dopaminergic neuron, and glioma), mouse (macrophages and neuroblasts), porcine (kidney), and human (lung, oral, colon, breast, cervical and epithelial cancer cells as well as fibroblasts, peripheral blood mononuclear, and endothelial and mesenchymal stem cells). Nanomaterials may induce autophagy via an oxidative stress mechanism, such as accumulation of damaged proteins and subsequent endoplasmic reticulum or mitochondrial stress [39, 89–92] and altering gene/protein expression and/or regulation, and interfering with the kinase-mediated regulatory cascades [93–103]. The increase in autophagic vacuoles in response to nanomaterials may be an adaptive cellular response. There is evidence that autophagy can selectively compartmentalize nanomaterials. In fact, nanoparticles are commonly observed within the autophagosome compartment, suggesting that activation of autophagy is a targeted exertion to sequester and degrade these materials following entrance into the cytoplasm [104]. It is possible that the cells might perceive nanomaterials as an endosomal pathogen or an aggregation-prone protein (both commonly degraded by the autophagy machinery). Recent evidence supports ubiquitination of nanomaterials directly or indirectly via colocalization with ubiquitinated protein aggregates, suggesting that cells may indeed select nanomaterials for autophagy through a pathway similar to invading pathogens [13, 98, 105]. Additionally, ubiquitinated proteins accumulate concomitantly with nanomaterial-induced autophagic vacuoles [106]. 

It is important to underlie that nanoscale was a significant factor in eliciting the autophagic response. Autophagy was not induced by quantum dots that had a tendency to aggregate to microscale particles into the cells [107]. Nanoscale size dependence was also reported for neodymium oxide nanoparticle, with larger particles inducing less autophagy [108]. Apparently, modifications of the surface properties might be able to alter the autophagy-inducing activity of the nanomaterials. Cationic PAMAM dendrimers elicited autophagy more than anionic ones *in vitro* [94]. Carbon nanotubes with carboxylic acid group could induce autophagy, while those functionalized with poly-aminobenzene sulfonic acid and polyethylene glycol groups were not [100]. Recently, it has been published that a short synthetic peptide, RE-1, binds to lanthanide-based nanocrystals, forms a stable coating layer on the nanoparticles surface, and significantly abolishes their autophagy-inducing activity. Furthermore, the addition of an arginine-glycine-aspartic acid motif to RE-1 enhances autophagy induced by lanthanide-based nanocrystals [109].

It is also possible that nanomaterials cause a state of autophagic dysfunction, correlated with a blockade of autophagy flux, and this may be involved in their mechanism of toxicity [110, 111]. Nanoparticles could give rise to autophagy dysfunction by overloading or directly inhibiting lysosomal enzymes or disrupting cytoskeleton-mediated vesicle trafficking, resulting in diminished autophagosome-lysosome fusion [112]. Nanoparticles could also directly affect lysosomal stability by inducing lysosomal oxidative stress, alkalization, osmotic swelling, or causing detergent-like disruption of the lysosomal membrane (see the complete review of Stern and colleagues [61] about this subject). Disruption in autophagosome trafficking to the lysosome has been implicated in several human pathologies, including cancer development and progression as well as neurodegenerative diseases. As exposure to airborne pollution has been associated with Alzheimer and Parkinson-like pathologies, and nanoparticles are the primary particle number and surface area component of pollution-derived particulates, Stern and Johnson have recently postulated a relationship between nanoparticle-induced autophagy dysfunction and pollution-associated neurodegeneration [113].

Several studies have been suggested also that the nanomaterial-induced autophagy dysfunction is correlated with mitochondrial damage [102, 114–118].

In the majority of the studies, autophagosome accumulation induced by nanomaterials treatment was associated with cell death, unfortunately the possibility of autophagy inhibition was not often investigated (the block of autophagy flux and autophagy induction both can determinate autophagosome accumulation) [119], and the mechanism of nanomaterial-induced autophagy accumulation in many cases is unclear.

**Figure 4:**
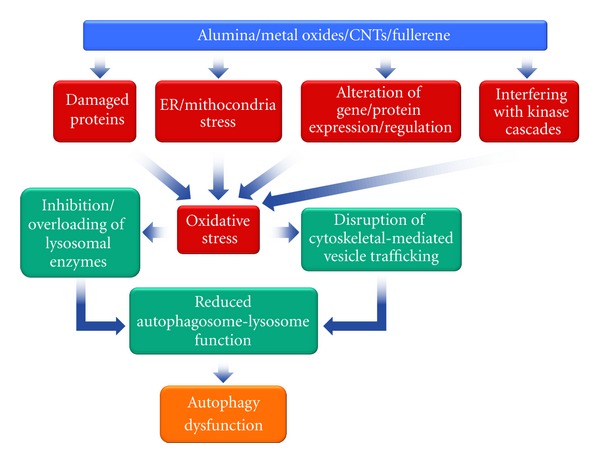


Interestingly, nanomaterials have been proposed also as tools to monitor autophagy [120, 121]. In conclusion, a growing body of the literature indicates that nanomaterials impact the autophagy pathways, then the possible autophagic response should be always taken into consideration in the development of novel nanomaterials systems (Figure 4). Moreover, further studies should be performed to clarify the molecular mechanisms underlying the interaction between nanomaterials and the autophagy machinery as well as to expand the knowledge of the implications and biological significance of this modulation.

## 5. Nanomaterials and Necrosis

Necrosis was, for a long time, considered as an accidental form of cell death, but in recent years several studies clarified that this process is regulated and may play a role in multiple physiological and pathological settings [122]. Several triggers can induce regulated necrosis, including alkylating DNA damage, excitotoxins, and the ligation of death receptors [38, 122]. Indeed, when caspases are genetically or pharmacologically inhibited, RIP1 (receptor-interacting protein kinase 1) and its homolog RIP3 are not degraded and engage in physical and functional interactions that ultimately activate the execution of necrotic cell death [38, 122]. It should be noted that RIP3-dependent and RIP1-independent cases of necrosis have been described, suggesting that there are several subprograms of regulated necrosis [38, 122–124]. In a genome-wide siRNA screen, Hitomi and colleagues elucidated the relationship between appotosis and necrosis pointing out that some components of the apoptotic pathway (e.g., the BH3-only protein Bmf) are also crucial in the necrotic machinery [125]. Moreover, recent studies provide evidence that apoptosis and necrosis are closely linked [126–128]. The term “necroptosis” has been used as a synonym of regulated necrosis, but it was originally introduced to indicate a specific case of necrosis, which is induced by death receptor ligation and can be inhibited by the RIP-1 targeting chemical necrostatin-1 [38, 122, 129].

In the literature, there are confused and inconsistent examples of necrosis induced by nanomaterials, because on one hand only the loss of cell viability is often evaluated without focalising into the cell death modalities and on the other hand, there are no single discriminative biochemical markers available yet. Moreover, it should not be underestimated that the induction of apoptosis in cell culture is inevitably followed by secondary necrosis, and this could lead to a misinterpretation of results. However, a recent study demonstrated that water-soluble germanium nanoparticles with allylamine-conjugated surfaces (4 nm) induce necrotic cell death that is not inhibited by necrostatin-1 in Chinese hamster ovary cells [130]. Although the mechanisms of ligand and surface chemistry, surface charge, and crystallinity-based toxicity are complex, studies are beginning to elucidate certain surface functional groups and properties that can effectively alter biological responses. In fact, the crystal structure, with the different forms, of nanomaterials can dictate its cytotoxic potential. Braydich-Stolle and coworkers identify that both size and crystal structure (rutile, anatase, and amorphous) of TiO_2_ nanoparticles affect the mechanism of cell death in mouse keratinocyte cell line [131]. They found that 100% anatase TiO_2_ nanoparticles induced necrosis in size-independent manner, whereas the rutile TiO_2_ nanoparticles elicited apoptosis. Pan and collaborators investigated the size-dependent cytotoxicity exhibited by gold nanoparticles (stabilized with triphenylphosphine derivatives) in several human cell lines. All cell types internalised gold nanoparticles and showed signs of stress. Smaller particles (<1.4 nm) were more toxic than their larger equivalents. However, 1.4 nm nanoparticles cause predominantly rapid cell death by necrosis, while closely related particles 1.2 nm in diameter affect predominantly apoptosis [132, 133]. Besides, it has been reported that small (10 nm) silver nanoparticles had a greater ability to induce apoptosis than other-sized ones (50 and 100 nm) in mouse osteoblastic cell line and induce necrosis in rat phaeochromocytoma cells [134]. The shape-dependent toxicity of polyaniline (PANI) nanomaterials with four different aspect ratios on human lung fibroblast cells was evaluated. The toxicity increased with decreasing aspect ratio of PANI nanomaterials; low aspect ratio PANI nanomaterials induced more necrosis than others [135]. Furthermore, the surface charge seems to be a major factor of how nanoparticles impact cellular processes. It has been demonstrated that charged gold nanoparticles induced cell death via apoptosis, whereas neutral nanoparticles caused necrosis [136]. Clearly, other parameters may influence the cell death modalities induced by nanomaterials, such as the dose or the time of exposure. Depending on the concentration, nano-C60 fullerene caused ROS-mediated necrosis (high dose), or ROS-independent autophagy (low dose) in rat and human glioma cell cultures [137]. The type of cell death induced by silver ions (Ag^+^) and silver nanoparticle coated with polyvinylpyrrolidone were also dependent on the dose and the exposure time, with Ag^+^ being the most toxic in a human monocytic cell line [138]. The silver nanoparticles concentrations required to elicit apoptosis were found to be much lower than the concentrations required for necrosis in human fibrosarcoma, skin, and testicular embryonal carcinoma cells [139, 140]. In conclusion, although the reports are often contradictory, the cell death appears roughly cell type, material composition, and concentration dependent. For instance, it has been reported that TiO_2_ (5–10 nm), SiO_2_ (30 nm), and MWCNTs (with different size: <8 nm, 20–30 nm, and >50 nm, but same length 0.5–2 *μ*m) induce cell-specific responses resulting in variable toxicity and subsequent cell fate in mouse fibroblasts and macrophages as well as telomerase-immortalized human bronchiolar epithelial cells. Precisely, the macrophages were very susceptible to nanomaterial toxicity, while fibroblasts are more resistant at all the treatments, whereas only the exposure of SiO_2_ and MWCNT (<8 nm) induce apoptosis in human bronchiolar epithelial cells. In the experimental conditions of this study, the investigated nanomaterials did not trigger necrosis [65]. In the same mouse macrophage cell line, it has been demonstrated that MWCNT (10–25 nm) and SWCNTs (1.2–1.5 nm) induced necrosis in a concentration-dependent manner [141]. CNTs have been demonstrated to induce both necrosis and apoptosis in human fibroblasts [142]. In contrast, Cui and co-workers found that SWNTs upregulate apoptosis-associated genes in human embryo kidney cells [143], and Zhu and colleagues showed that MWCNTs induce apoptosis in mouse embryonic stem cells [144], while Pulskamp and collaborators assert that commercial CNTs do not induce necrosis or apoptosis in rat macrophages [145]. Recently, a multilevel approach, including different toxicity tests and gene-expression determinations, was used to evaluate the toxicity of two lanthanide-based luminescent nanoparticles, complexes with the chelating agent EDTA. The study revealed that these nanomaterials induced necrosis in human lymphoblasts and erythromyeloblastoid leukemia cell lines, while no toxicity was observed in human breast cancer cell line. Moreover, no *in vivo* effects have been observed. The comparative analysis of the nanomaterials and their separated components showed that the toxicity was mainly due to the presence of EDTA [146].

The knowledge advances concerning the molecular characterization of necrosis will make necessary more precise and accurate studies to confirm the ways in which nanomaterials might cause necrotic death.

## 6. Nanomaterials and Pyroptosis

Pyroptosis described the peculiar death of macrophages infected by *Salmonella typhimurium *[147]. Several other bacteria triggering this atypical cell death modality have been identified. Pyroptosis neither constitutes a macrophage-specific process nor a cell death subroutine that only results from bacterial infection. Pyroptotic cells can exhibit apoptotic and/or necrotic morphological features. The most distinctive biochemical feature of pyroptosis is the early caspase-1 activation associated with the generation of pyrogenic mediators, such as Interleukin-1*β* (IL-1*β*) [38].

Recently, it has been shown that the exposure of macrophages (both a mouse macrophage cell line and primary human alveolar macrophages) to carbon black nanoparticles resulted in inflammasome activation as defined by cleavage of caspase-1 to its active form and downstream IL-1*β* release. The carbon black nanoparticles-induced cell death was identified as pyroptosis through the inhibition of caspase-1 and pyroptosis by specific pharmacological inhibitors. The authors showed that, in this setting, TiO_2_ particles did not induce pyroptosis or significantly activate the inflammasome [148]. In contrast, it has been shown that nano-TiO_2_ and nano-SiO_2_, but not nano-ZnO (zinc oxide) and carbon nanotubes, induced inflammasome activation but not cell death in murine bone marrow-derived macrophages and human macrophages cell line. Although the caspase-1 cleavage and IL-1*β* release was induced, the inflammation caused by nanoparticles was largely caused by the biological effect of IL-1*α* [149]. This apparent discrepancy could be explained considering the different concentration and kind of nanomaterials used in these studies; moreover, it is possible that different macrophages perform differently in response to nanomaterials. Future studies should address this issue. However, the identification of pyroptosis as a cellular response to carbon nanoparticles exposure is novel and relates to health impacts of carbon-based particulates.

## 7. Conclusions and Perspectives

The continued expansion of the nanotechnology field requires a thorough understanding of the potential mechanisms of nanomaterial toxicity for proper safety assessment and identification of exposure biomarkers. With increasing research into nanomaterial safety, details on the biological effects of nanomaterials have begun to emerge. The nanomaterials intrinsic toxicity has been attributed to their physicochemical characteristics, that is, their smallness and the remarkably large surface area per unit mass and high surface reactivity. In fact, their type, composition and modifications, size, shape, and surface charge should be considered. However, the complex death paradigms may also be explained by activation of different death pathways in a context-dependent manner. *In vitro* experiments could be influenced by a cell type-specific response, and ones *in vivo* could be affected by the animal species and the model used or by pharmacokinetic parameters (administration, distribution, metabolism, etc.). Moreover, the dose, concentrations, and the time of exposure of a nanomaterial employed are essential. In effect, the efficiency of cellular uptake of nanomaterials and the resultant intracellular concentration may determine the cytotoxic potential. Elucidating the molecular mechanisms by which nanosized particles induce activation of cell death signalling pathways will be critical for the development of prevention strategies to minimize the cytotoxicity of nanomaterials. Unfortunately, in the literature, there are many conflicting data; the most plausible reason is certainly the discrepancy of nanomaterials and experimental models engaged. Although some authors have recently alerted colleagues on these issues [3, 5, 8, 9, 150–152], it has not yet been put in place a guideline, generally accepted by the scientific community in the field, to address these matters. In fact, harmonization of protocols for material characterization and for cytotoxicity testing of nanomaterials is needed. In addition, parallel profiling of several classes of nanomaterials, combined with detailed characterization of their physicochemical properties, could provide a model for safety assessment of novel nanomaterials [153]. During the past decade, owing to major technological advances in the field of combinatorial chemistry in addition to the sequencing of an ever increasing number of genomes, high-content chemical and genetic libraries have become available, raising the need for high-throughput screening (HTS) and high-content screening (HCS) approaches. In response to this demand, multiple conventional cell death detection methods have been adapted to HTS/HCS, and many novel HTS/HCS-amenable techniques have been developed [37, 154]. In the last years, several authors started to study the nanotoxicity with this tools and highlighted the potential of these approaches [9, 60, 75, 155–161]. An overall aim should identify HTS/HCS assays that can be used routinely to screen nanomaterials for interaction with the cell death modalities system. HTS/HCS may accelerated the analysis on a scale that commensurates with the rate of expansion of new nanomaterials but in any case is a first validation step, then it remains to confirm whether the same identified mechanisms *in vitro* are responsible for their *in vivo* toxicity. In conclusion, a multilevel-integrated uniform and consistent approach should contemplate for nanomaterial toxicity characterization.

In spite of the recent advances in our understanding of cell death mechanisms and associated signalling networks, much work remains to be done before we can fully elucidate the toxicological behaviour of the nanomaterials as well as understand their participation in the determination of cell fate. More and accurate results are needed for apoptosis, autophagy, and necrosis induction by nanomaterials; further studies are necessary to test if the novel strategic targets identified could be affected either directly or indirectly by nanomaterials. Moreover, no data are present in the literature concerning the nanomaterials exposure and other forms of cell death including anoikis, entosis, parthanatos, netosis, and cornification. For example, although numerous studies have been performed on keratinocytes, none of these has rated cornification, a cell death subroutine restricted to keratinocytes and functionally linked to the generation of the stratum corneum of the epidermis [38]. It will be of considerable interest to establish whether these various cell death modalities are associated with the intent of identifying a structure-activity relationship and delineating the mechanisms by which these interactions occur. In addition to the established paradigms of nanomaterials toxicity, the study of their interactions with the death signalling pathways could potentially have many important human pathological outcomes, including cancer, metabolic disorders, and neurodegenerative disorders.

## Abbreviations

Ag^+^: Silver ions
ATG: Autophagy-related gene
Bcl-2: B-cell lymphoma 2
BH3: Bcl-2 homology domain 3
BID: BH3-interacting domain death agonist
Bmf: Bcl-2-modifying factor
CNTs: Carbon nanotubes
CoCr: Cobalt-chrome
DNA: Deoxyribonucleic acid
EDTA: Ethylenediaminetetraacetic acid
ER: Endoplasmic reticulum
FDA: Food and Drug Administration
HCS: High-content screening
HTS: High-throughput screening
IL: Interleukin
MOMP: Mitochondrial outer membrane permeabilization
MWCNTs: Multiwall carbon nanotubes
NADPH: Nicotinamide adenine dinucleotide phosphate
NCCD: Nomenclature Committee on Cell Death
PAMAM: Cationic polyamidoamine
PANI: Polyaniline
PEG-PE: Poly(ethylene glycol)-phosphoethanolamine
RIP: Receptor-interacting protein kinase
RNA: Ribonucleic acid
RNS: Reactive nitrogen species
ROS: Reactive oxygen species
SiO_2_: Silicon dioxide
siRNA: Small interfering RNA
SWCNTs: Single-walled carbon nanotubes
tBID: Truncated BID
TiO_2_: Titanium dioxide
ZnO: Zinc oxide.
